# Describing Temperament in an Ungulate: A Multidimensional Approach

**DOI:** 10.1371/journal.pone.0074579

**Published:** 2013-09-10

**Authors:** Katharina L. Graunke, Gerd Nürnberg, Dirk Repsilber, Birger Puppe, Jan Langbein

**Affiliations:** 1 Ethology Unit, Institute of Behavioural Physiology, Leibniz Institute for Farm Animal Biology (FBN), Dummerstorf, Germany; 2 Institute of Genetics and Biometry, Bioinformatics and Biomathematics Unit, Leibniz Institute for Farm Animal Biology (FBN), Dummerstorf, Germany; 3 Faculty of Agricultural and Environmental Sciences (AUF), PHENOMICS office, University of Rostock, Rostock, Germany; 4 Faculty of Agricultural and Environmental Sciences (AUF), Behavioural Sciences, University of Rostock, Rostock, Germany; Institut Pluridisciplinaire Hubert Curien, France

## Abstract

Studies on animal temperament have often described temperament using a one-dimensional scale, whereas theoretical framework has recently suggested two or more dimensions using terms like “valence” or “arousal” to describe these dimensions. Yet, the valence or assessment of a situation is highly individual. The aim of this study was to provide support for the multidimensional framework with experimental data originating from an economically important species (*Bos taurus*). We tested 361 calves at 90 days *post natum* (dpn) in a novel-object test. Using a principal component analysis (PCA), we condensed numerous behaviours into fewer variables to describe temperament and correlated these variables with simultaneously measured heart rate variability (HRV) data. The PCA resulted in two behavioural dimensions (principal components, PC): novel-object-related (PC 1) and exploration-activity-related (PC 2). These PCs explained 58% of the variability in our data. The animals were distributed evenly within the two behavioural dimensions independent of their sex. Calves with different scores in these PCs differed significantly in HRV, and thus in the autonomous nervous system’s activity. Based on these combined behavioural and physiological data we described four distinct temperament types resulting from two behavioural dimensions: “neophobic/fearful – alert”, “interested – stressed”, “subdued/uninterested – calm”, and “neoophilic/outgoing – alert”. Additionally, 38 calves were tested at 90 and 197 dpn. Using the same PCA-model, they correlated significantly in PC 1 and tended to correlate in PC 2 between the two test ages. Of these calves, 42% expressed a similar behaviour pattern in both dimensions and 47% in one. No differences in temperament scores were found between sexes or breeds. In conclusion, we described distinct temperament types in calves based on behavioural and physiological measures emphasising the benefits of a multidimensional approach.

## Introduction

Differences in behaviour between animals can be caused by a range of environmental and state-dependent factors, e.g., sex, age, reproductive status, or environment. However, not all differences can be explained by physiological states or environmental factors; the remaining differences may therefore reveal the strategy an individual employs to act with and react to environmental stimuli [1–3]. This individual strategy is described as temperament and is thought to be innate and consistent over time and in different situations [4–9]. Besides temperament there are other terms used in this context, e.g., personality, individuality or coping style [6,10–12]. Réale et al. [6] provide a summary of the different terms and their definitions depending on the author(s), which clearly shows how arbitrary the distinctions between the different terms are. They conclude that the two most common terms, temperament and personality, are often artificially distinguished. Therefore, they understand these two terms as synonyms, which we are in accordance with. Although being consistent over time and situations, temperament should not be imagined as a fixed and completely inflexible construct, but rather as an adjustable tool for adaptation to exterior circumstances during individual ontogeny [13]. Very early in life, temperament seems to be rather flexible [14,15], while later on it is maintained more and more rigidly [13]. However, depending on genetics and epigenetics, the starting point is different for each individual.

In the literature, original research on non-human animals often describes temperament on a one-dimensional scale using expressions such as “proactive – reactive”, “aggressive – non-aggressive”, “bold – shy”, etc. (e.g., [11,16,17]). Human psychology and thereon based recent theoretical framework on temperament in non-human animals, however, mostly argue for two or more dimensions using terms such as “valence”, “arousal” or “activity” to describe the different dimensions [6,18–22]. Following their arguments, different temperament types can be located in a circumplex model as a linear combination of these dimensions. Therefore, two or more dimensions are more likely to reflect the entire nature of temperament or personality in non-human animals than one dimension. Especially, the valence or perception of a situation is highly individual, yet most important to an animal’s welfare [23]. Veissier et al. [23] point out that while exterior conditions like housing system, quality of diet, etc. loose no importance to animal welfare, one mandatorily needs to take into account the valence of the animals themselves, when seriously trying to evaluate an animal’s welfare.

Naturally, questionnaires on the perception of different situations that can be answered in personality research in human psychology are impossible in non-human animals; therefore, one must include measures of physiological or neurophysiological activation revealing information about the probable perception and processing of a test situation by an individual. The analysis of cardio-vascular measurements has been found to be a suitable approach for determining the activity of the autonomous nervous system in the study of temperament [24–26]. The cardiac vagal tone represents parasympathetic nervous activity at the level of the heart and derives from heart rate variability (HRV). HRV measures can be calculated from beat to beat changes in heart rate (R-R interval) in the electrocardiogram. The cardiac vagal tone has been suggested as a psychophysiological marker of internal regulation and of certain aspects of psychological adjustment in humans and animals [27,28]. Changes in the length of consecutive R-R-intervals reflect differential activation of the two branches of the autonomous nervous system [29,30] and can therefore give an understanding of a test subject’s perception or its valence of a situation. Common variables of HRV measures in the time domain are the heart rate in beats per minute (HR in bpm), the root mean square of successive differences (RMSSD in ms), the standard deviation of all R-R-intervals (SDNN in ms) and the ratio of RMSSD and SDNN (RMSSD/SDNN). Table 1 provides a description of the effects of the autonomous nervous system on these measures.

**Table 1:** Influence of the autonomous nervous system on measures of heart rate variability.

HRV measures	Influence of the autonomous nervous system	Consequences on HRV measure
HR	Additive and non-additive effects of PNS and SNS	HR decreases, when PNS activity increases and/or SNS activity decreases
RMSSD	Only influenced by PNS	RMSSD increases, when PNS activity increases
SDNN	PNS and SNS act synergetically	SDNN increases mainly, when SNS activity increases, but is also influenced by PNS activity
RMSSD/SDNN	PNS and SNS affect measure antagonistically	RMSSD/SDNN increases, when PNS activity increases and/or SNS activity decreases

Measures of heart rate variability (HRV), branches of the autonomous nervous system that influence the HRV measures, and their consequences on the respective measure (after [29]); PNS = parasympathetic nervous system, SNS = sympathetic nervous system

The aim of this study was to develop a description of temperament in young cattle (*Bos taurus*) by analysing their behaviour and simultaneously measured heart rate variability during a standard behaviour test. To test for stability over time, we conducted the test at two ages on an additional, small sample size. Taking this combined multidimensional approach based on experimental original data, we intended to provide foundational support for the theoretical framework suggesting two or more dimensions in animal temperament.

## Animals, Materials and Methods

### 2.1: Animals and housing

We tested 361 calves (175 male, 186 female) of the F_2_-generation of a running breeding project (Holstein Friesian × Charolais cross breeding) with 90 dpn (± 3 dpn, days *post natum*). All calves were bred via embryo transfer into unrelated Holstein Friesian heifers as recipient mothers and were born and tested between 2004 and 2010. The calves were kept in various small groups of up to nine animals of similar age, apart from their recipient mothers from day one. Pens had a size of 6 x 7 m and were covered with deep litter. Until 90 dpn, the calves were not subject to any other experiment, and handling did not exceed routine handling by the animal keepers except in the case of animals requiring treatment for sickness.

After weaning, the animals were weighed at 111 dpn (± 3 dpn). The weight at the day of the experiment was calculated with the help of the average daily weight gain from birth to weaning and the exact age at the experiment. On average, the calves weighed 118 kg (range: 74-159 kg, SD ± 14 kg) at the day of the experiment.

We further tested each 20 calves (10 male, 10 female) of the founder breeds Holstein Friesian and Charolais at 91 dpn (± 3 dpn) and a second time at 197 dpn (± 12 dpn) to evaluate stability of temperament over time and to detect possible breed differences. The calves were purchased from breeders and arrived at our facilities two days after birth at the latest. They were housed in the same barn as the crossbreeds and male and female calves were housed together until the second test had been conducted. These calves were born and tested between 2008 and 2012. Due to the early death of one male Charolais calf, there were 19 Charolais calves tested at 91 dpn. At the second test age, one male Charolais calf became extremely distressed during testing and risked serious injury. The test was terminated; thus there is data of 18 (8 male, 10 female) Charolais calves at 197 dpn.

### 2.2: Experimental procedure

The behaviour test was performed in an open field of 9.6 × 4.0 m in size, which was unknown to the calves prior to testing. It was divided into four segments of 2.4 × 4.0 m each. After allowing the test animal to acclimatise to the open field for 10 min, a novel-object test was conducted with a traffic pylon of 0.5 m height as novel object. It was let down into the outer segment, which was the farthest from where the calf stood (Figure S1). We chose this test as it is known to provoke behaviour, which correlates with behaviour during other tests [31,32] or with social cues [31]. The novel-object test lasted for 10 min. During the test, behaviour was live-recorded using the observation software tool The Observer 5.0 (Noldus, The Netherlands). Of in total 438 behaviour test sessions, 428 were conducted by three experienced observers whose observation highly correlated during a 90 min-test session (Pearson’s Rho 0.973, p < 0.001). The residual 10 behaviour test sessions were conducted by three other experienced observers. Recorded behaviours with their definitions and type of recording are listed in Table 2. For further analysis, the latency of the behaviours an individual did not show during the 10 min behaviour test was set to the maximum time of 600 s (10 min).

**Table 2:** Definition of live-recorded behaviours.

Behaviour	Type of recording	Definition
Contact with novel object (contact)	D, F, L	Physical contact with any part of the body with the novel object or sniffing the novel object while being closer than 0.1 m to it
Inactivity	D	At least three legs touch the ground, no forward movement
Exploration	D, L	Sniffing or licking the wall or floor of the open field
Grooming	D	Calf licking or scratching itself with one hind leg
Activity	D, L	Max. 3 legs touch the ground, forward movement
Running	D	Max. 2 legs touch the ground, fast forward movement
Vocalisation	F	Any kind of sound the calf makes
Change of segment	F	Leaving one segment and entering another with at least the forelegs
Habitation in segment where the novel object is placed (object segment)	D, L	With at least the forelegs in the segment in which the novel object is placed
Habitation in segment next to segment where the novel object is placed (object neighbouring segment)	L	With at least the forelegs in the segment next to the segment in which the novel object is placed

Definition and type of recording of the behaviours live-recorded during the novel-object test; D = duration (total time in s), F = frequency, L = latency (time in s until behaviour was first shown).

### 2.3: Heart rate variability (HRV)

To measure the heart beat activity during the test, we applied a heart monitor system (Polar S810i, Polar Electro, Oy, Finland). The calves were fitted with flexible belts with two integrated electrodes and a transmitter for wireless transmission of the R-R-interval data series to a separate storage device. The two electrodes were placed on the left side of the most cranial part of the chest behind the forelegs: one next to the sternum, and the other behind the scapula. The coat under the electrodes was shaved and a conductive gel was used for better electrical conductivity. Prior to the beginning of the experiment, calves were fitted the belts and were then left alone with their pen mates in their home pen to gain base measurements. After 30 min, they were led into the open field for acclimatisation and testing. Later on, the R–R data series were transferred to a computer and corrected when necessary using Polar Precision Performance SW version 4.03 (Polar Electro, Oy, Finland) with the standard set-up. The curves were divided into 5-min intervals and an error correction of up to 10% per interval was accepted. In further processing of the data, neither differences between two R-R-intervals larger than 150 ms nor identical values of five or more consecutive R-R-intervals were accepted. A program developed with LabView 2009 version 9.0 (National Instruments Germany GmbH, Munich, Germany) detected complete 1-min intervals in the base measurements (starting 5 min after the experimenters left the barn) and the test, and calculated HR, RMSSD, SDNN, and RMSSD/SDNN for each complete 1-min interval (see [30] for the exact calculation of the variables, see Introduction for an explanation of the variables). When there were at least seven complete 1-min intervals per base measurement and per test (134 male, 138 female), the program further determined the mean of the first seven values of HR, RMSSD, SDNN and RMSSD/SDNN. The differences between test and base measurements of HR, RMSSD and SDNN and the ratio of test and base measurement of RMSSD/SDNN were used for further analyses. Later analysis showed that it was completely coincidental and independent of the animals’ behaviour during the novel-object test (e.g., running or activity duration), which calves did and which did not have complete HRV measures. Therefore, there is no further discussion of this fact.

All procedures involving animal handling and treatment were approved by the Committee for Animal Use and Care of the Ministry of Agriculture, the Environment and Consumer Protection of the federal state Mecklenburg-Vorpommern, Germany.

### 2.4: Statistical analysis

All statistical analyses were performed using SAS 9.3, SAS Institute Inc., USA. In a preliminary analysis, we checked for the influence of sex and weight on all behaviours and HRV measures using a one-way analysis of covariance model (ANCOVA, The GLM Procedure) with the fixed factor sex and the co-variable weight.

As main analysis, we performed a Principal Component Analysis (PCA), which described the relationship between new (latent) principal components (PC) and our 15 behaviours. A PCA is used to condense several correlated measures into a smaller number of principal components. The loadings of each measure on a principal component represent the correlation between the component and this measure. i.e., the loadings reflect the importance of each measure for the component. One of the main assumptions for using PCA or Factor Analysis for an analysis of data is a suitable correlation between all included measurements. A measure for this sampling adequacy (MSA) of the correlation matrix is the Kaiser-Meyer-Olkin criterion (KMO). As Budaev [33] mentioned “correlation matrices with KMO < 0.5 are entirely inappropriate whereas those with KMO below 0.6-0.7 must be treated with caution”. We decided to use a PCA instead of a Factor Analysis, because many of our behaviour measurements were non-normally distributed [33], and because no a-priori theory or model exists [34]. The PCA was conducted with The FACTOR Procedure with the following parameter settings: method=PRIN, prior=ONE, rotation=VARIMAX. As input data set, we used a correlation matrix of all pairwise correlations of our 15 behavioural measures applying the non-parametric Spearman’s rank correlation test (using The CORR Procedure), because some of the behaviours were not continuous and/or normally distributed (Table S1). One crucial point when using a PCA is the choice of the final number of extracted PCs [33]. Several methods are available for this decision. We performed four methods: Kaiser’s number of eigenvalues > 1 [35], Cattell’s scree-test [36], Horn’s Parallel test [37] and Velicer’s Minimum Average Partial (MAP) test [38]. For the Parallel test and MAP test, we applied the SAS syntaxes provided by O’Connor [39]. We decided for two PCs in the final PCA calculation, since three of these methods led to a two PC solution (except number of eigenvalues > 1). Corresponding PC scores for each calf were finally calculated with The SCORE Procedure. These scores were further used to determine score classes and to identify the calves with differing behaviour.

The influence of these score classes and sex on the HRV measures was tested by a two-way analysis of variance model (ANOVA, The MIXED Procedure) with the fixed factors score class, sex and their interaction. Post hoc tests were performed with a Tukey-Kramer correction for multiple testing.

The PC scores of the calves of the founder breeds (Holstein Friesian, Charolais) were calculated with The SCORE Procedure using the resulting loadings from the crossbreeds as it is no use to perform a PCA on such a low number of animals [40]. The influence of breed and sex on the score class was calculated with a two-way ANOVA (The MIXED Procedure) with the fixed factors breed, sex and their interaction. To analyse the stability of the scores over time, we applied Spearman’s rank correlation test on scores of the two test ages (The CORR Procedure). For all analyses, we defined the significance level at 0.05 and treated p-values between 0.05 and 0.1 as tendency.

## Results

### 3.1: Behaviour and heart rate variability

Descriptive statistics of the recorded behaviours of 361 crossbreed calves in the novel-object test are shown in Table S2. The HRV measures of 272 of these calves during base measurement and novel-object test are presented in Table S3. Weight had a significant influence on grooming duration with lighter calves grooming longer than heavier calves (F = 4.25, p = 0.040), but had no influence on any other behaviour. Sex had a significant influence on grooming duration and latency of activity (F = 11.02, p < 0.001; F = 4.15, p = 0.042) and tended to have an influence on change of segment (F = 2.73, p = 0.099), where male calves groomed longer, had a lower latency to show activity and changed segments less often than female calves. Weight had a significant influence on RMSSD/SDNN with lighter calves having higher measures than heavier calves (F = 5.41, p = 0.021), but none on any other HRV measure. Sex had an influence on HR, SDNN and RMSSD/SDNN (F = 4.35, p = 0.038; F = 7.30, p = 0.007; F = 8.20, p = 0.005), with male calves having a lower HR, lower SDNN and higher RMSSD/SDNN than female calves.

During the novel-object test, calves of the two breeds Charolais and Holstein Friesian did not differ in their behaviour except for the duration of running (F = 4.25, p = 0.046), with Charolais calves running longer than Holstein Friesian calves (least square mean 5.1 s vs. 1.4 s). Accordingly, Charolais calves had a higher HR and lower RMSSD than Holstein Friesian calves (F = 12.6, p = 0.001; F = 5.1, p = 0.031). Since SDNN did not differ between the breeds, the RMSSD/SDNN differed accordingly (F = 6.4, p = 0.016). None of the HRV measures differed between the breeds during base measurement.

### 3.2: Principal component analysis

The loadings of the behaviours in the two PCs gained from the PCA of the novel-object test are shown in Table 3. The loadings rated “excellent” (greater than 0.71 and lower than -0.71) and “very good” (greater than 0.63 and lower than -0.63) were accepted as explanatory variables [40]. PC 1 was most influenced by behaviours occurring in the novel-object context such as contact duration or the time spent close to the object, and PC 2 was most influenced by behaviours in context with the exploration of the open field (but not the novel object) and the inactivity of the animals. The measure of sampling adequacy (MSA) with 0.833 was “meritorious” [41] and our data therefore appropriate for PCA analysis [33]. The two PCs explained 46.8% and 11.2%, respectively, of the variation in the data.

**Table 3:** Principal component loadings of the behaviours.

Behaviour	PC 1	PC 2
Contact-D	**0.76457**	0.05006
Contact-F	**0.83250**	0.12250
Contact-L	**-0.89613**	-0.13959
Inactivity-D	-0.41347	**-0.85549**
Exploration-D	0.15037	**0.82661**
Exploration-L	-0.19767	*-0.63679*
Grooming-D	-0.08038	0.42876
Activity-D	0.56716	0.61371
Activity-L	-0.49598	-0.21244
Running-D	0.47287	0.34835
Vocalisation-F	0.38216	0.07442
Change of segment-F	*0.70109*	0.51393
Object segment -L	**-0.87210**	-0.15841
Object segment-D	**0.83888**	0.12061
Object neighbouring segment -L	**-0.73723**	-0.22699

Loadings of the behaviours in principal component (PC) 1 and PC 2 gained from the principal component analysis of the novel-object test; loadings above 0.71 in bold type, loadings above 0.63 in italics (cf. [40] loadings above 0.71 rated “excellent”, loadings above 0.63 rated “very good”); D = duration (total time in s), F = frequency, L = latency (time in s until behaviour was first shown).

**Figure 1:**
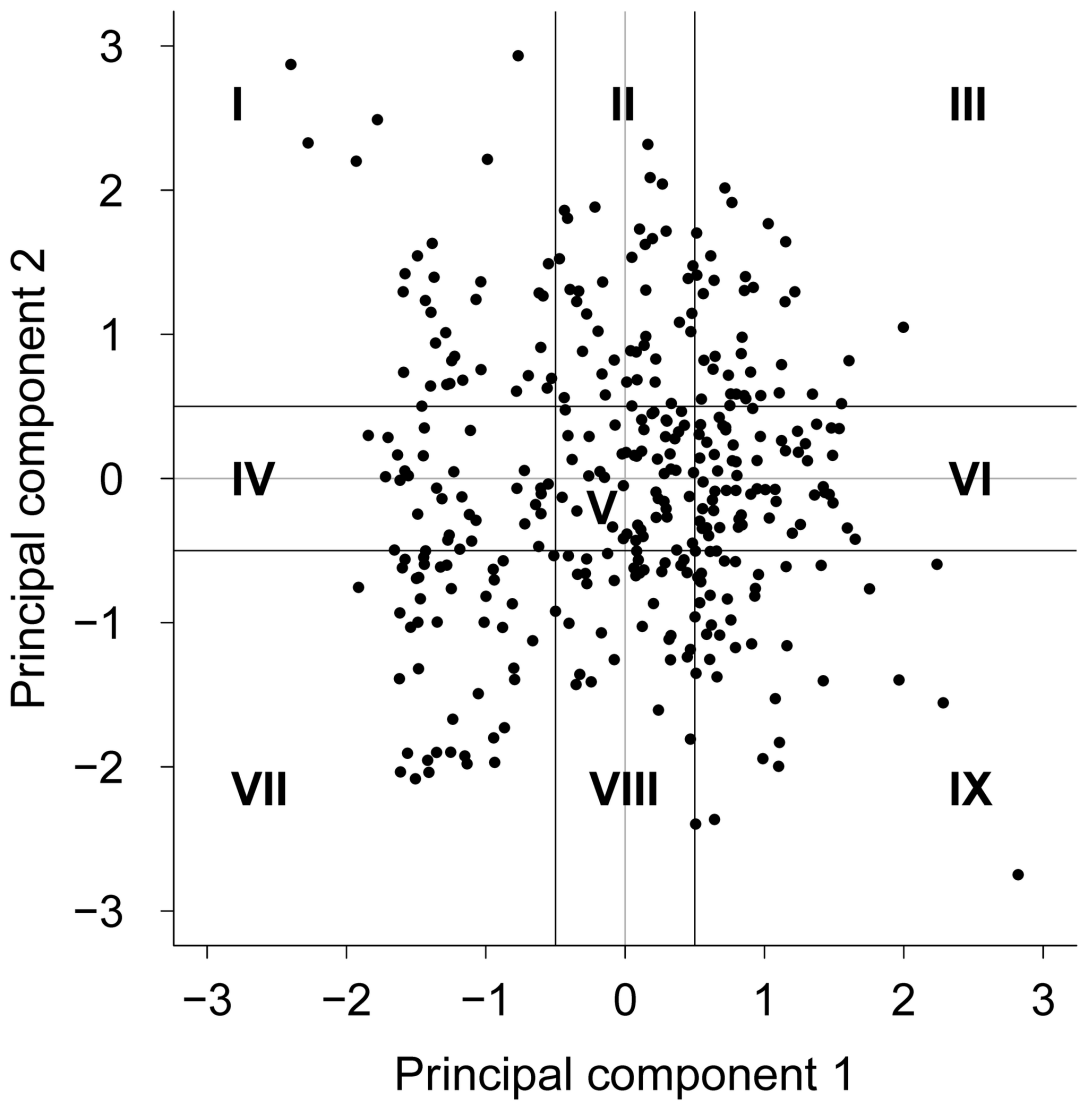
Scores plot of the crossbreed calves. Scores plot of 361 crossbreed calves gained from the standardised original data of the novel-object test and the respective loadings in the two PCs (Table 3), including the classification into nine score classes, numbered with Roman numerals; a range of ± 0.5 SD from the zero line was defined as threshold for the intermediate level.

For each animal, the scores in PC 1 and PC 2 were calculated from their standardised original data and the respective loadings as presented in Table 3. To distinguish animals from one another by their temperament, we divided the animals into score classes (SC) according to the level of their scores in the two PCs (as suggested by Mendl et al. [20]). We defined the intermediate level of the scores at ± 0.5 SD around the zero line to identify calves not showing distinct behaviour in one or both PCs. Using this procedure, we received nine SCs (Figure 1). A plot with the scores of all 361 crossbreed calves is shown in Figure 1, where each dot represents one calf. The distribution of the male and female calves in the SCs is presented in Table 4.

**Table 4:** Distribution of the calves on the score classes.

SC	Male	Female
I	20	13
II	23	16
III	12	22
IV	16	17
V	22	28
VI	28	32
VII	20	22
VIII	15	18
IX	19	18

Distribution of crossbreed calves subdivided by sex on the score classes (SC) in the novel object test.

Calves of SC IX had long contact to the novel object and hardly explored the open field, while those of SC I explored the open field a long time, made little or very late contact to the novel object and were highly active. However, using information from only behaviour made it difficult or impossible to describe some of the SCs and to understand the animals’ temperament. We therefore analysed the heart rate variability measures for differences based on SC, sex and the interaction of SC and sex.

### 3.3: Development of temperament types (TT)

**Figure 2:**
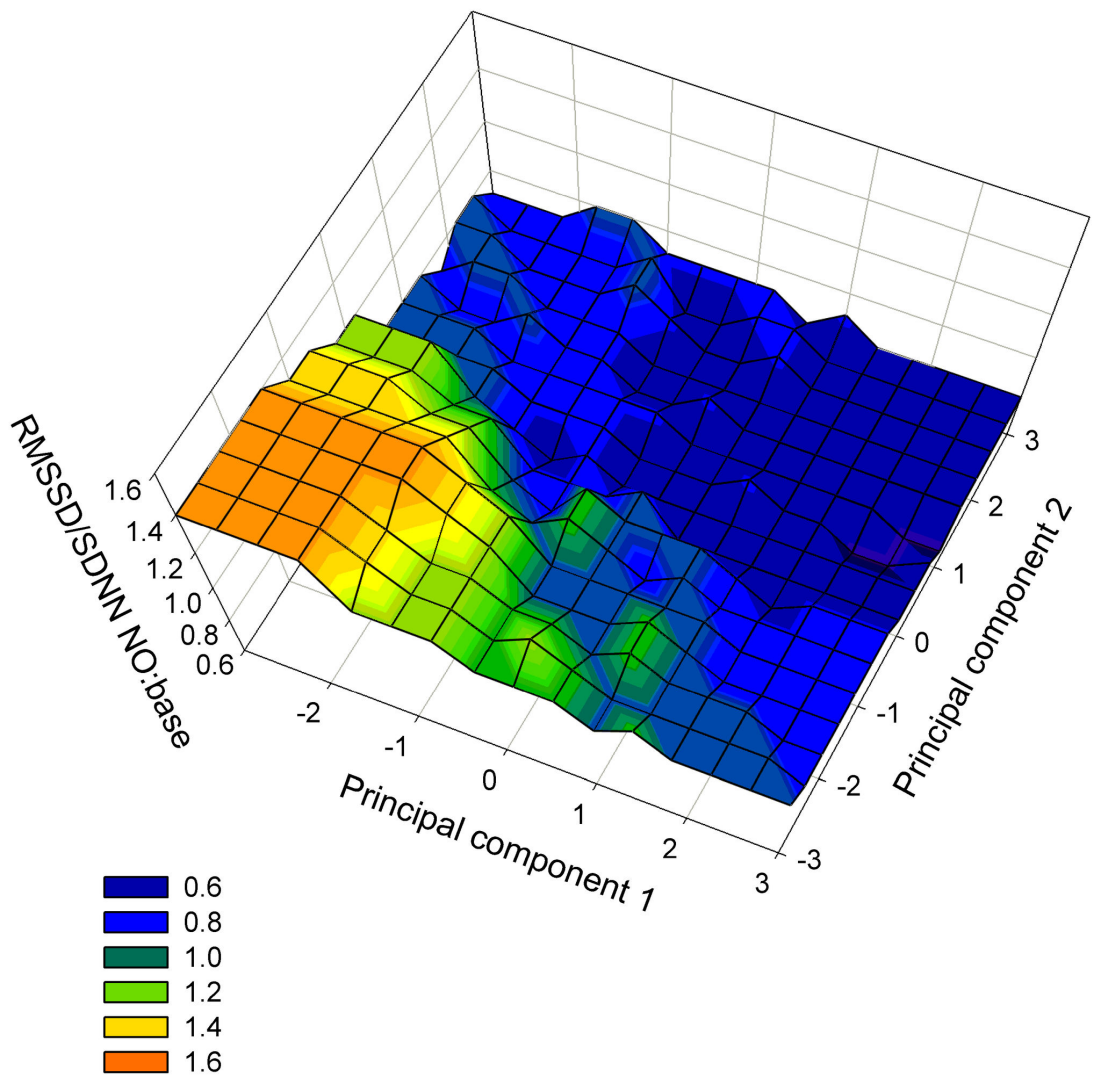
3D scores plot including the RMSSD/SDNN-ratio. Smoothed 3D scores plot of 361 crossbreed calves during the novel-object test (NO) with the ratio of RMSSD/SDNN between NO and base measurement as the third dimension; colour spectrum from dark blue (strongly sympathetically activated) to red (strongly parasympathetically activated), smoother “running median”, bandwidth method “nearest neighbours”, and sampling proportion 0.100 (SigmaPlot 10.0, SysStat Software Inc., USA).

The interaction of SC and sex had no significant influence on the changes in any of the four HRV variables (HR:F = 0.52, p = 0.840; RMSSD: F = 0.34, p = 0.948; SDNN: F = 1.36, p = 0.212; RMSSD/SDNN: F = 0.48, p = 0.871). Sex had no significant influence (RMSSD: F = 0.04, p = 0.834; SDNN: F = 0.14, p = 0.707; RMSSD/SDNN: F = 0.66, p = 0.417) except for a tendency for HR change, where the female calves tended to have a higher increase in HR during the behaviour test compared to the male calves (t = 1.92; p = 0.056). SC had a significant influence on the changes in HR, SDNN and RMSSD/SDNN (HR:F = 5.19; SDNN: F = 6.64; RMSSD/SDNN: F = 5.04; all p < 0.001), but not on RMSSD (RMSSD: F = 0.73, p = 0.666). Most reliable information about the balance between sympathetic and parasympathetic nervous system is gained from the RMSSD/SDNN. The least square means, their standard error and the 95% confidence interval of the RMSSD/SDNN-ratio between test and base measurement are shown in Table 5. A value larger than 1.00 indicates a shift towards the parasympathetic nervous system during the test, while a value smaller than 1.00 indicates a shift towards the sympathetic nervous system. Confidence intervals not embracing 1.00 indicate a significant shift of the autonomous nervous system during the test compared to the base measurement. During the test, animals of SC III and VI showed a significant shift towards the sympathetic nervous system, whereas in those of SC I, II, IV, V, VIII, and IX the balance between the sympathetic and parasympathetic nervous system did not change (Table 4, Figure 2). Calves of SC VII had a 46% higher RMSSD/SDNN-ratio during the novel-object test compared to the base measurement (Table 4), i.e. they were on average strongly parasympathetically activated while calves of SC III and VI were sympathetically activated during the novel-object test. Therefore, we could describe the most distinct TT (SC I, III, VII, and IX) by characteristic terms for the displayed behaviour and the activated parts of the autonomous nervous system: “neophobic/fearful – alert” (SC I), “interested – stressed” (SC III), “subdued/uninterested – calm” (SC VII), and “neoophilic/outgoing – alert” (SC IX; Figure 2).

**Table 5:** Least square means of the nine score classes of the RMSSD/SDNN-ratio.

SC	LSM	SE	95% CI
I	0.93	0.10	0.73-1.12
II	0.92	0.08	0.75-1.08
III	0.82	0.09	0.64-1.00
IV	1.05	0.08	0.89-1.22
V	0.92	0.08	0.76-1.07
VI	0.83	0.06	0.70-0.96
VII	1.46	0.08	1.31-1.62
VIII	1.04	0.08	0.88-1.21
IX	0.97	0.09	0.80-1.15

Least square means (LSM), standard error (SE), and 95% confidence interval (CI) of the nine score classes (SC) of the RMSSD/SDNN-ratio; 1.00 indicates no change in the ratio during the novel-object test compared to the base measurement.

### 3.4: Stability over time

Each individual - intermediate or distinct – has its own specific temperament. Therefore, to evaluate stability of the scores, one needs to take all individuals into account.

#### 3.4.1: Stability within the two-dimensional space

The distribution of the Charolais and Holstein Friesian calves was even within the scores plot (Figure S2) revealing no breed differences in the scores (PC 1: F = 0.13, p = 0.724; PC 2: F = 0.03, p = 0.867). During the repetition of the test procedure at 197 dpn (days *post natum*) 42.1% of the calves scored within 1 SD around their score at 90 dpn, 44.7% scored between 1–2 SD around their first score and 13.2% scored farther than 2 SD from their first score. Neither sex (F = 0.00, p = 0.973) nor breed (F = 0.22, p = 0.639) or interaction between sex and breed (F = 1.51, p = 0.228) influenced the difference in the score between the first and second test age. The animals scoring within 1 SD (n = 16) at both test ages showed various directions in the changes (Figure S2A), while 15 of 17 animals scoring between 1–2 SD from their first score showed a greater change in one PC (> 1 SD) and only a small change in the other (< 1 SD, Figure S2B). Of these 17 calves, 11 showed a lower score in PC 2 at the second test age, and 9 simultaneously showed a change of less than 1 SD in PC 1. Of the 17 calves, 5 showed a greater score (> 1 SD) in PC 1 at 197 dpn than at 90 dpn, 3 of which simultaneously showed a change of less than 1 SD in PC 2. Of the 5 calves scoring farther than 2 SD from the first score, one showed a small change (< 1 SD) in PC 1 and two showed a small change (< 1 SD) in PC 2 (Figure S2C).

#### 3.4.2: Stability within each principal component

**Figure 3:**
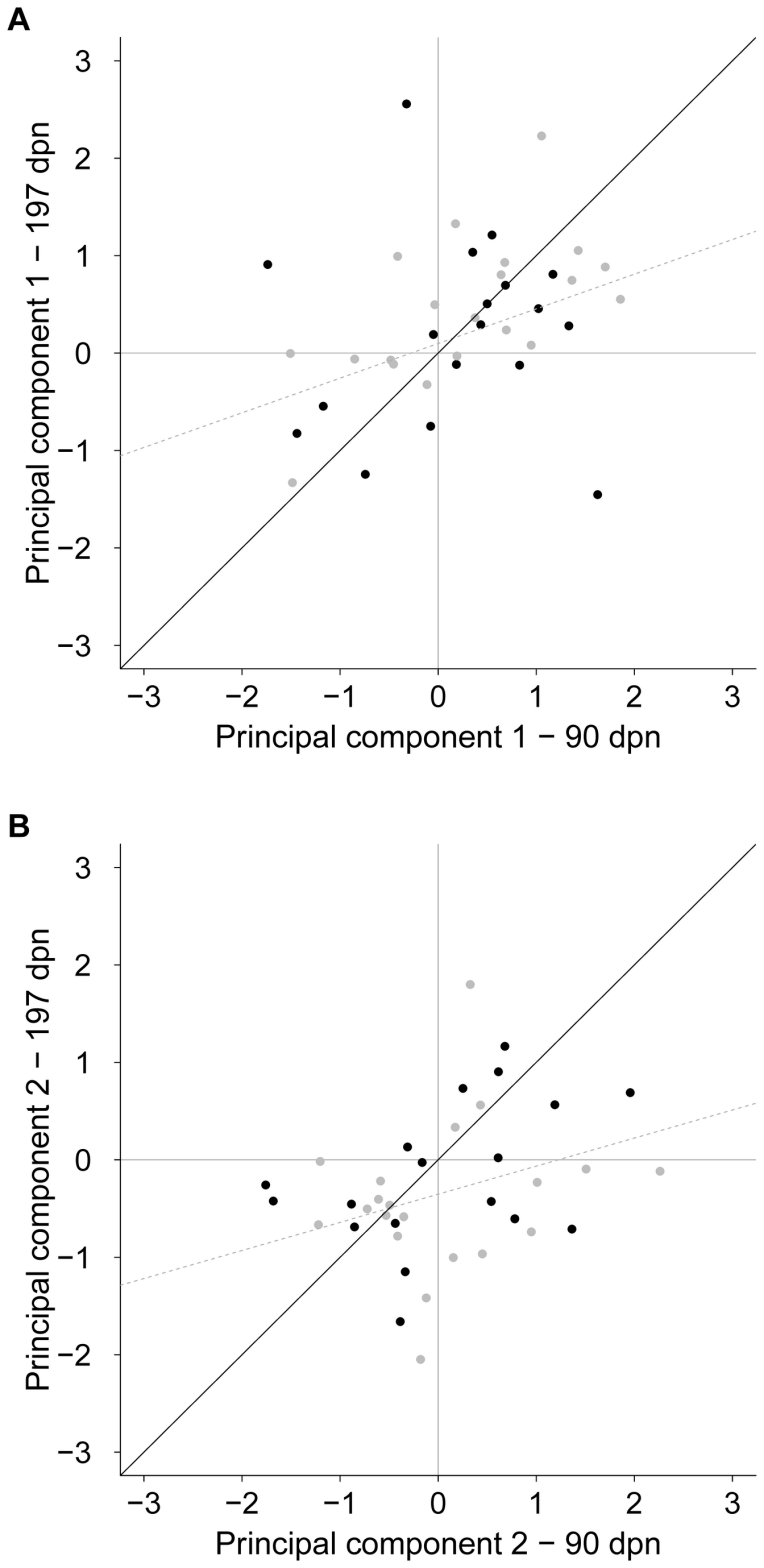
Score changes between the test ages separately for each principal component (PC). Changes in behavioural score between the test ages presented separately for (A) PC 1 and (B) PC 2 of 18 Charolais calves (black dots) and 20 Holstein Friesian calves (grey dots); solid black line marks 100% stability over time, dashed grey line marks the trend line.

Figure 3 shows the stability over the two test ages separately for each PC with the solid black line indicating 100% stability. Scores of PC 1 significantly correlated between the test ages (r = 0.36, p = 0.028; Figure 3A) and scores of PC 2 tended to correlate between 90 and 197 dpn (r = 0.29, p = 0.079; Figure 3B). In the same SC scored 21.1% of the calves, 39.5% stayed within the same score level in PC 1 (e.g., change from SC I to SC IV) and 10.5% in PC 2 (e.g., change from SC VII to SC IX). 28.9% of the calves changed SC on both score levels. However, no animal changed from SC I to SC IX or from SC III to SC VII and vice versa, meaning no animal changed from one extreme SC to the opposite SC.

## Discussion

Studies on animal temperament have often described temperament on a one-dimensional scale [11,16,17] while theoretical framework has suggested the necessity of two or more dimensions [6,18–20,42]. Only recently have studies started to present behavioural data condensed into two to four dimensions; however, except for Meager et al. [43] without characterising individual animals’ temperament [44–46]. To our knowledge, the presented study is the first to support the claims of the above named theoretical framework with original data based on non-human mammals. Using a multivariate analysis we could condense numerous behaviours to fewer variables to display different dimensions of temperament, support the interpretation of the behaviours with physiological data and describe individual animals’ temperament types. In context of cognitive enrichment, the approach of combining behavioural analyses with physiological data has successfully been used to test the animals’ validation of the enrichment [47,48]. The high number of several hundred tested individuals in the presented study lets us provide foundational support for the theory of multidimensional temperament in non-human animals.

With an MSA of 0.833, the data was suited “meritoriously” [41] for the conducted PCA, which was therefore absolutely appropriate [33]. The fact that PC 2 of this analysis explains a much smaller percentage of variance in our data leads to the assumption that the high-loading behaviours in PC 2 explain less variance in the behaviour of the calves than the high-loading behaviours in PC 1. Therefore, we conclude that most variance in the data was caused by the animals’ reactions to the novel object. It is unlikely that the calves would treat the open field as novel object, because they had time to acclimatise and to explore the open field prior to their exposure to the novel object. Réale et al. [6] define the category “exploration-avoidance” as independent of the category “boldness-shyness”. Equally in our analysis, the exploration of the open field (shyness-boldness) loaded high in one PC, whereas seeking contact to and “exploring”’ the novel object (exploration-avoidance) loaded high in the other PC. The exploration of the open field could, therefore, describe the activity of the calves. The data from the RMSSD/SDNN ratio clearly demonstrated that animals with similar scores in PC 1 did not necessarily respond similarly in their physiological reaction; this result is consistent for similar scores in PC 2. Hence, the perception or valence of the test situation was most likely different in different individuals [24], although they might have had similar scores in one of the PCs.

The multidimensional depiction of temperament or personality originates from human psychology [21,49] and was first implemented theoretically for animals by Koolhaas et al. and Mendl et al. [19,20]. If we compare the two-tier model suggested by Koolhaas et al. [19] with our plot, place it at a second level on top of our scores plot and turn it clockwise 45°, so the arousal dimension is aligned with our RMSSD/SDNN ratio results, we can see that our descriptions for the different TT are similar to those suggested by them. Also, if we compare our scores plot with the core affect model suggested by Mendl et al. [20] and turn it clockwise 45°, so that the arousal dimension in Mendl et al.’s [20] plot is aligned to our RMSSD/SDNN ratio results, and if we assume that the valence dimension in their plot remains orthogonal to its arousal dimension, we see that our descriptions for the different TT are similar to those suggested by them. We must emphasise, though, that neither of our PCs exactly represents any of the dimensions suggested by the two reviews [19,20]. Yet, one might argue that PC 1 might be consistent with the valence or coping dimension. One could assume that individuals approaching the novel object perceive the situation rather positively (hence have a positive valence of the situation) and are coping proactively; however, this cannot be scientifically supported with the by us conducted test alone. When attempting to fit our two dimensions “contact to novel object-related” and “exploration-activity-related” into the five categories of temperament traits defined by Réale et al. [6], we find PC 1 to be congruent to category 2: exploration-avoidance, reaction to among others novel objects. PC 2, though, cannot be easily fitted into this model. It could reflect the general activity level of the test calf (category 3: activity), but the test situation could also be perceived as risky by the calves (category 1: shyness-boldness, this measurement can interfere with exploration-avoidance). The sociability of the animal (category 5: sociability, seeking presence of or avoiding conspecifics, by exploring the open field for a way back to the home pen) or a combination of the above mentioned categories are also possible explanations for PC 2.

When we used the loadings generated with data from 361 crossbreed animals on data of the animals from the two founder breeds at the same age and in a test repetition 4 months later, we received a similarly even distribution of those animals on the scores as of the scores of the crossbreeds. PC 1, which explained almost half of the variance in our data, correlated between the two test ages 90 and 197 dpn. Many animals were close to the 100% stability line in this PC. This result confirmed findings of other work, where individual differences were consistent over time in various species, some of which were tested at early ages [4,8,32,50–52]. PC 2 did not show similarly good results in terms of stability. As many animals with larger differences in that PC showed less exploration of the open field and more inactivity during the second test, one could argue for increased habituation to the open field or the test situation.

Interestingly, we could not find any differences in the scores between calves of the two breeds Charolais and Holstein Friesian. With the exception of Charolais calves running longer than Holstein Friesian calves, there were no differences between the breeds in the original data or the HRV base measurement. Breed differences in various behaviour tests have been reported; occasionally including relatively high heritability scores [53–56]. However, the conducted tests measured the reaction of cattle towards humans, which has been reported to be more influenced by management system than by breed [57]. We consider it likely, that the temperament traits measured in the presented study are evolutionary so profoundly important [6,20], that their expression does not differ between different breeds of the same species. Still, we cannot exclude the possibility that the two cattle breeds might develop differently in the measured temperament traits when they age past 7 months. Various temperament traits, though, have been reported to be already stable at the early age of 6-8 months in cattle and horses [32,50–52].

## Conclusion

By using a principal component analysis to condense behaviours measured in calves in a novel-object test to two principal components (PC) and by correlating these PCs with heart rate variability measures, we could successfully describe four distinct temperament types that differed in behaviour and activity of the autonomous nervous system: “neophobic/fearful – alert”, “interested – stressed”, “subdued/uninterested – calm”, and “neoophilic/outgoing – alert”. During a repetition of the conducted novel-object test 4 months after the first test, more than 40% of the calves showed a similar behaviour pattern. In the remaining calves, the change was owed to a larger change in only one PC in nearly four-fifth of the animals. The novel object-related behaviours satisfactorily correlated between the two test ages. No differences in temperament scores could be found between sexes or breeds. Finally, we could describe distinct temperament types in calves based on behavioural and physiological measures emphasising the benefits of a multidimensional approach. The temperament-dependent assessment of a situation by the animals themselves should further be considered when trying to evaluate the housing and welfare of animals living under human care.

## Supporting Information

Figure S1 **Open field.** Diagram of the open field (9.6 × 4.0 m) where the novel-object test was performed; circles indicate the alternative standing positions for the novel object, segment size 2.4 × 4.0 m.
(TIFF)

Figure S2 **Score changes between the test ages.** Changes in behavioural scores of 18 Charolais calves (black dots) and 20 Holstein Friesian calves (grey dots); arrow heads indicate the score of the same individual at the second test age of 197 dpn; for clarity (A) shows arrows for the 16 individuals scoring within 1 SD around their score at 90 dpn, (B) shows arrows for the 17 individuals scoring between 1–2 SD around their first score, and (C) shows arrows for the 5 individuals scoring farther than 2 SD from their first score.
(TIF)

Table S1 Correlation matrix with Spearman correlation coefficients of the 15 behaviours of the crossbreed calves during the novel-object test; D = duration (total time in s), F = frequency, L = latency (time in s until behaviour was first shown).
(DOCX)

Table S2 Mean ± SD, median, minimum, and maximum of behaviours of crossbreed calves during the novel-object test; duration and latency in s; D = duration (total time in s), F = frequency, L = latency (time in s until behaviour was first shown).
(DOCX)

Table S3 Mean ± SD, median, minimum and maximum of heart rate (HR in bpm), RMSSD (ms), SDNN (ms), and RMSSD/SDNN of crossbreed calves during base measurement in the home pen and during the novel-object test.
(DOCX)
