# Supplementary figures and images for: Describing Temperament in an Ungulate: A Multidimensional Approach
Source: PLoS One. 2013 Sep 10;8(9):e74579. doi: 10.1371/journal.pone.0074579 (PMC3769396; doi:10.1371/journal.pone.0074579)

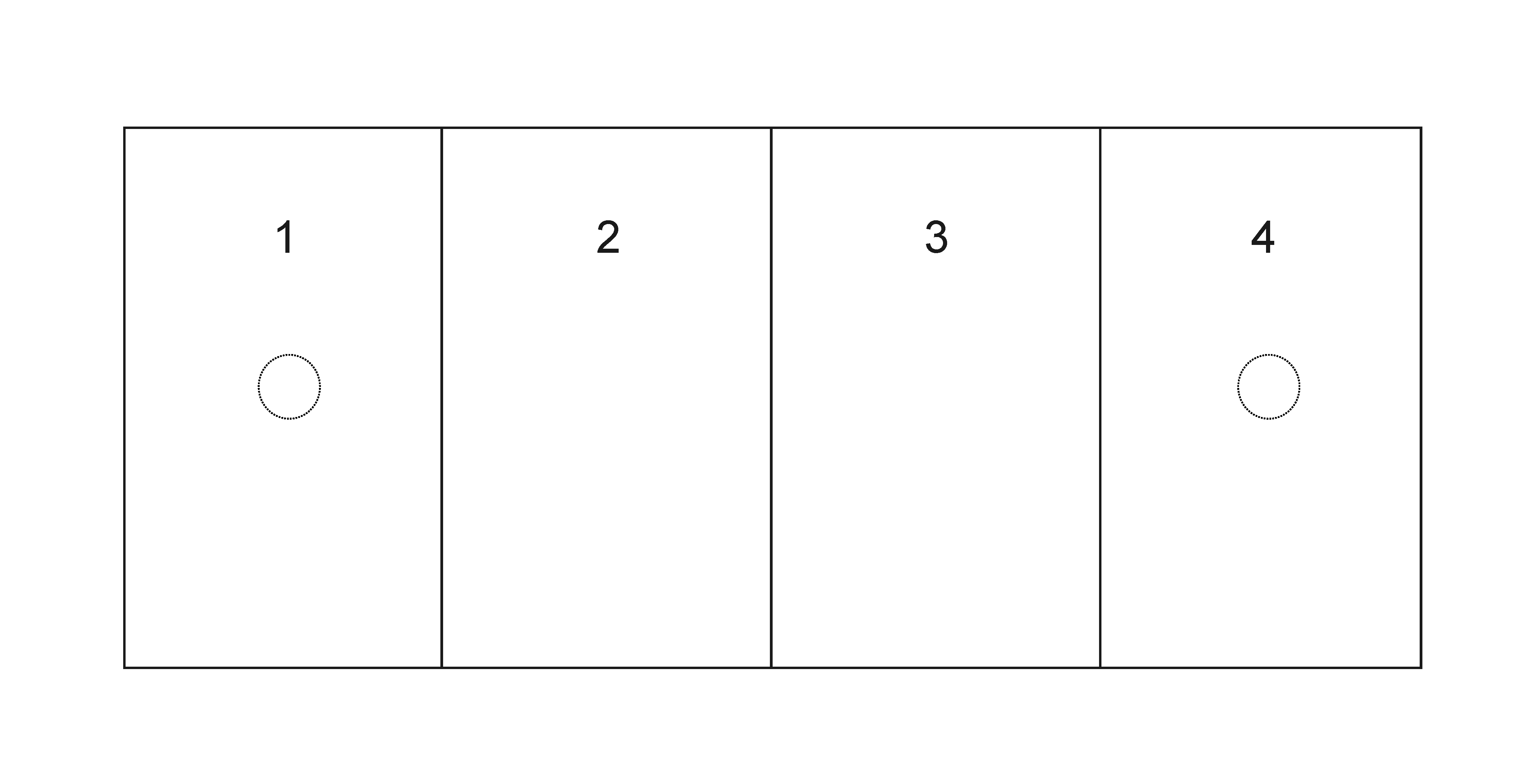

Supplement: Figure S1 — Open field. Diagram of the open field (9.6 × 4.0 m) where the novel-object test was performed; circles indicate the alternative standing positions for the novel object, segment size 2.4 × 4.0 m. (TIFF) [file pone.0074579.s001.tiff]
