# Supplementary material for: Describing Temperament in an Ungulate: A Multidimensional Approach
Source: PLoS One. 2013 Sep 10;8(9):e74579. doi: 10.1371/journal.pone.0074579 (PMC3769396; doi:10.1371/journal.pone.0074579)
Supplement: Table S1 — Correlation matrix with Spearman correlation coefficients of the 15 behaviours of the crossbreed calves during the novel-object test; D = duration (total time in s), F = frequency, L = latency (time in s until behaviour was first shown). (DOCX) [file pone.0074579.s003.docx]

**Table S1.** Correlation matrix of the 15 behaviours.

|  |  | 1 | 2 | 3 | 4 | 5 | 6 | 7 | 8 | 9 | 10 | 11 | 12 | 13 | 14 |
| --- | --- | --- | --- | --- | --- | --- | --- | --- | --- | --- | --- | --- | --- | --- | --- |
| 1 | Contact-D |  |  |  |  |  |  |  |  |  |  |  |  |  |  |
| 2 | Contact-F | 0.825 |  |  |  |  |  |  |  |  |  |  |  |  |  |
| 3 | Contact-L | -0.618 | -0.698 |  |  |  |  |  |  |  |  |  |  |  |  |
| 4 | Inactivity-D | -0.388 | -0.461 | 0.481 |  |  |  |  |  |  |  |  |  |  |  |
| 5 | Exploration-D | 0.257 | 0.320 | -0.262 | -0.820 |  |  |  |  |  |  |  |  |  |  |
| 6 | Exploration-L | -0.192 | -0.278 | 0.304 | 0.494 | -0.491 |  |  |  |  |  |  |  |  |  |
| 7 | Grooming-D | 0.068 | -0.012 | -0.047 | -0.252 | 0.185 | -0.153 |  |  |  |  |  |  |  |  |
| 8 | Activity-D | 0.424 | 0.511 | -0.557 | -0.802 | 0.447 | -0.382 | 0.145 |  |  |  |  |  |  |  |
| 9 | Activity-L | -0.247 | -0.309 | 0.436 | 0.329 | -0.163 | 0.281 | -0.092 | -0.396 |  |  |  |  |  |  |
| 10 | Run-D | 0.279 | 0.368 | -0.416 | -0.474 | 0.274 | -0.186 | 0.022 | 0.427 | -0.205 |  |  |  |  |  |
| 11 | Vocalisation-F | 0.172 | 0.259 | -0.306 | -0.210 | 0.135 | -0.043 | 0.009 | 0.253 | -0.141 | 0.287 |  |  |  |  |
| 12 | Change of segment-F | 0.469 | 0.609 | -0.652 | -0.724 | 0.432 | -0.362 | 0.038 | 0.836 | -0.399 | 0.667 | 0.349 |  |  |  |
| 13 | Object segment-L | -0.555 | -0.623 | 0.926 | 0.478 | -0.241 | 0.333 | -0.059 | -0.553 | 0.438 | -0.430 | -0.288 | -0.646 |  |  |
| 14 | Object segment-D | -0.425 | -0.483 | 0.698 | 0.455 | -0.232 | 0.306 | -0.140 | -0.519 | 0.624 | -0.359 | -0.237 | -0.597 | 0.747 |  |
| 15 | Object neighbouring segment-L | 0.703 | 0.764 | -0.719 | -0.472 | 0.304 | -0.268 | 0.014 | 0.501 | -0.351 | 0.365 | 0.272 | 0.576 | -0.700 | -0.545 |

**Table S1.** Correlation matrix with Spearman correlation coefficients of the 15 behaviours of the crossbreed calves during the novel-object test; D = duration (total time in s), F = frequency, L = latency (time in s until behaviour was first shown).
