# Supplementary material for: Describing Temperament in an Ungulate: A Multidimensional Approach
Source: PLoS One. 2013 Sep 10;8(9):e74579. doi: 10.1371/journal.pone.0074579 (PMC3769396; doi:10.1371/journal.pone.0074579)
Supplement: Table S2 — Mean ± SD, median, minimum, and maximum of behaviours of crossbreed calves during the novel-object test; duration and latency in s; D = duration (total time in s), F = frequency, L = latency (time in s until behaviour was first shown). (DOCX) [file pone.0074579.s004.docx]

**Table S2.** Behaviours during the novel-object test.

| Behaviour | Mean | SD | Median | Min | Max |
| --- | --- | --- | --- | --- | --- |
| Contact-D | 26.3 | 37.0 | 18.1 | 0.0 | 321.9 |
| Contact-F | 3.2 | 2.7 | 3.0 | 0 | 17 |
| Contact-L | 285.5 | 215.9 | 225.3 | 6.0 | 600.0 |
| Inactivity-D | 428.1 | 90.5 | 424.7 | 130.0 | 600.0 |
| Exploration-D | 69.1 | 51.1 | 59.5 | 0.0 | 250.6 |
| Exploration-L | 142.1 | 134.4 | 104.9 | 2.0 | 600.0 |
| Grooming-D | 5.8 | 12.7 | 0.0 | 0.0 | 114.8 |
| Activity-D | 80.5 | 47.9 | 74.9 | 0.0 | 317.5 |
| Activity-L | 60.8 | 95.7 | 24.5 | 0.0 | 600.0 |
| Run-D | 8.7 | 14.3 | 2.9 | 0.0 | 111.0 |
| Vocalisation-F | 15.3 | 14.9 | 11.0 | 0 | 76 |
| Change of segment-F | 17.5 | 12.7 | 15.0 | 0 | 65 |
| Object segment-L | 262.3 | 215.5 | 196.4 | 0.0 | 600.0 |
| Object segment-D | 174.6 | 189.1 | 123.7 | 0.0 | 591.4 |
| Object neighbouring segment-L | 131.9 | 110.4 | 103.9 | 0.0 | 600.0 |

**Table S2.** Mean ± SD, median, minimum, and maximum of behaviours of crossbreed calves during the novel-object test; duration and latency in s; D = duration (total time in s), F = frequency, L = latency (time in s until behaviour was first shown).
