# Supplementary material for: Describing Temperament in an Ungulate: A Multidimensional Approach
Source: PLoS One. 2013 Sep 10;8(9):e74579. doi: 10.1371/journal.pone.0074579 (PMC3769396; doi:10.1371/journal.pone.0074579)
Supplement: Table S3 — Mean ± SD, median, minimum and maximum of heart rate (HR in bpm), RMSSD (ms), SDNN (ms), and RMSSD/SDNN of crossbreed calves during base measurement in the home pen and during the novel-object test. (DOCX) [file pone.0074579.s005.docx]

**Table S3.** Heart rate variability measures during base measurement and novel-object test.

| HRV measures | Base measurement | | | | | Novel-object test | | | | |
| --- | --- | --- | --- | --- | --- | --- | --- | --- | --- | --- |
|  | Mean | SD | Median | Min | Max | Mean | SD | Median | Min | Max |
| HR | 113.9 | 16.4 | 113.4 | 70.2 | 186.4 | 125.0 | 17.9 | 125.0 | 68.0 | 190.5 |
| RMSSD | 7.86 | 7.00 | 5.64 | 2.14 | 68.06 | 8.71 | 6.01 | 6.39 | 2.93 | 37.25 |
| SDNN | 23.86 | 9.26 | 22.23 | 4.92 | 62.27 | 31.70 | 10.34 | 30.76 | 10.33 | 70.67 |
| RMSSD/SDNN | 0.35 | 0.19 | 0.28 | 0.12 | 1.15 | 0.31 | 0.18 | 0.25 | 0.13 | 1.16 |

**Table S3.** Mean ± SD, median, minimum and maximum of heart rate (HR, bpm), RMSSD (ms), SDNN (ms), and RMSSD/SDNN of crossbreed calves during base measurement in the home pen and during the novel-object test.
